# Effect of Epicatechin against Radiation-Induced Oral Mucositis: *In Vitro* and *In Vivo* Study

**DOI:** 10.1371/journal.pone.0069151

**Published:** 2013-07-18

**Authors:** Yoo Seob Shin, Hyang Ae Shin, Sung Un Kang, Jang Hee Kim, Young-Taek Oh, Keun Hyung Park, Chul-Ho Kim

**Affiliations:** 1 Department of Otolaryngology, School of Medicine, Ajou University, Suwon, Republic of Korea; 2 Department of Otorhinolaryngology-Head and Neck Surgery, National Health Insurance Corporation Ilsan Hospital, Goyang, Republic of Korea; 3 Department of Pathology, School of Medicine, Ajou University, Suwon, Republic of Korea; 4 Department of Radiation Oncology, School of Medicine, Ajou University, Suwon, Republic of Korea; Winship Cancer Institute of Emory University, United States of America

## Abstract

**Purpose:**

Radiation-induced oral mucositis limits the delivery of high-dose radiation to head and neck cancer. This study investigated the effectiveness of epicatechin (EC), a component of green tea extracts, on radiation-induced oral mucositis *in vitro* and *in vivo*.

**Experimental Design:**

The effect of EC on radiation-induced cytotoxicity was analyzed in the human keratinocyte line HaCaT. Radiation-induced apoptosis, change in mitochondrial membrane potential (MMP), reactive oxygen species (ROS) generation and changes in the signaling pathway were investigated. *In vivo* therapeutic effects of EC for oral mucositis were explored in a rat model. Rats were monitored by daily inspections of the oral cavity, amount of oral intake, weight change and survival rate. For histopathologic evaluation, hematoxylin-eosin staining and TUNEL staining were performed.

**Results:**

EC significantly inhibited radiation-induced apoptosis, change of MMP, and intracellular ROS generation in HaCaT cells. EC treatment markedly attenuated the expression of p-JNK, p-38, and cleaved caspase-3 after irradiation in the HaCaT cells. Rats with radiation-induced oral mucositis showed decreased oral intake, weight and survival rate, but oral administration of EC significantly restored all three parameters. Histopathologic changes were significantly decreased in the EC-treated irradiated rats. TUNEL staining of rat oral mucosa revealed that EC treatment significantly decreased radiation-induced apoptotic cells.

**Conclusions:**

This study suggests that EC significantly inhibited radiation-induced apoptosis in keratinocytes and rat oral mucosa and may be a safe and effective candidate treatment for the prevention of radiation-induced mucositis.

## Introduction

Radiotherapy has become increasingly important for the treatment of head and neck squamous cell carcinoma [1], [2]. Oral mucositis, associated with both radiation and chemotherapy, is a very common, painful, and dose-limiting toxicity, with an incidence that can be as high as 90% [3]. Radiation-induced oral mucositis may be accompanied by altered taste, pain, dry mouth, decreased appetite, and even ulceration, and thus can lead to decreased nutritional uptake [3], [4]. Oral mucositis, especially in its ulcerative form, increases morbidity, mortality, and costs of cancer therapy [5]. The presence of ulcerative oral mucositis in granulocytopenic patients often leads to systemic infections, and even to fatal consequences. Oral mucositis may significantly detract from the quality of life of cancer patients undergoing radiotherapy or chemotherapy. Furthermore, oral mucositis can cause clinicians to interrupt the therapy of patients, which is quite undesirable as it can limit the success of therapy. Therefore, there have been a lot of efforts to mitigate radiation-induced oral mucositis. [6] [7] [8] [9] [10] [11] [12] [13], [14] [3], [15] Despite this, no intervention has yet been completely successful in preventing radiation-induced oral mucositis [16].

Radiation-induced oral mucositis develops because radiation therapy can interfere with the maturity and cellular growth of epithelial cells, causing changes to normal turnover and cell death. Irradiated epithelial, endothelial, and connective tissue cells in the buccal mucosa release free radicals, modified proteins, and proinflammatory cytokines, including interleukin-1B, prostaglandins, and tumor necrosis factor [17]. Biochemical changes such as an increase in the generation of reactive oxygen species (ROS) and depletion of glutathione can occur in irradiated cells prior to the loss of surface membrane integrity [18]. Studies using ROS scavengers such as vitamin E, amifostine, and N-acetylcysteine have provided evidence of the importance of ROS as an early trigger leading to oral mucositis [19], [20]. Another promising way to find a radioprotector is to find natural products with antioxidant actions. Among the various phytochemicals, green tea polyphenols showed strong anti-oxidative property and are considered to be powerful ROS scavenging agents [21]. The goal of the present study was to investigate the *in vitro* radio-protective effects of epicatechin (EC), a component of green tea extracts, on radiation-induced cell death and associative mechanisms in the HaCaT human keratinocyte line and the *in vivo* efficacy of EC in rats.

## Materials and Methods

### Cell Lines and Irradiation Conditions

Human keratinocytes (HaCaT cell line) were obtained from the American Type Culture Collection (ATCC, Manassas, VA, USA). HaCaT cells were maintained in high glucose Dulbecco’s modified Eagle’s medium (DMEM; Gibco, Grand Island, NY, USA) containing 10% fetal bovine serum (FBS; Gibco). The cells were cultured in a humidified incubator at 37°C in an atmosphere containing 5% CO_2_. Cells were prepared in 6-well or 24-well plates and irradiated with single doses of 20 Gy. A single 20 Gy dose was delivered by opposed photon beams at a rate of 2 Gy/min using the LINAC, 6MV (21EX; Varian, Palo Alto, CA, USA).

### Cell Viability Assay

HaCaT cells were pre-treated with various concentrations of EC (0–200 µM) for 1 h, and then washed twice with cold PBS and prepared in 24-well plates. A single dose of 20 Gy was applied. To determine cell viability, the HaCaT cells were seeded in 96-well plates at densities of 5×10^3^ cells/well in 1 ml complete medium after being exposed to radiation. At 5 days interval after irradiation, 3-(4,5-dimethyl-thiazol-2-yl)-2. 5-diphenyl-2H-tetrazolium bromide (MTT; Sigma-Aldrich, St. Louis, MO, USA) was added to 40 µl of the cell suspension for 4 h. After three washes with phosphate buffered saline (PBS, pH 7.4), the insoluble formazan product was dissolved in dimethyl sulfoxide (DMSO). The optical density (OD) of each culture well was measured using a microplate reader (Bio-Tek, Winooski, VT, USA) at 540 nm.

### Terminal Deoxynucleotidyl Transferase (TdT)-mediated dUTP-biotin Nick End Labeling (TUNEL) Assay

HaCaT apoptosis was determined by the TUNEL method using an *in situ* cell detection kit (Roche Molecular Biochemicals, Mannheim, Germany). The cells were exposed to medium with EC (100 µM) only, radiation only (20 Gy), or radiation (20 Gy) plus EC (100 µM). Cells were processed as described previously [22]. The stained cells were analyzed using a fluorescence microscope (Carl Zeiss, Oberkochen, Germany). TUNEL-positive cells were manually counted under a 200X magnification field in triplicate.

### Annexin V-fluorescein Isothiocynate (FITC)/Propidium Iodide (PI) Double Staining

The quantitative analysis of apoptotic cell death caused by irradiation was performed using the FITC Annexin V Apoptosis Detection Kit II (Becton Dickinson, Franklin Lakes, NJ, USA). The cells were stained with following the manufacturer’s protocol. HaCaT cells were plated at 1×10^6^ cells/well in a 6-well plate, incubated for 16 h, and then treated with EC (100 µM) only, radiation only (20 Gy), or radiation (20 Gy) plus EC (100 µM). The stained cells were analyzed by fluorescence-activated cell sorting (FACS) using an ARIA3 apparatus (BD Biosciences, San Jose, CA, USA) equipped with WinMDI 2.9 Software.

### Mitochondrial Membrane Potential Assay

The mitochondrial membrane potential (MMP) of intact cells was measured by flow cytometry with the lipophilic cationic probe 5,5,V,6,6 V-tetrachloro-1,1 V 3,3 V-tetra ethylbenzimidazolcarbocyanine iodide (JC-1; Molecular Probes). The cells were exposed to medium with EC (100 µM) only, radiation only (20 Gy), or radiation (20 Gy) plus EC (100 µM). Cells were processed as described previously [22]. The change in MMP was measured by flow cytometry and fluorescence microscopy at 72 h after irradiation.

### Measurement of Intracellular ROS Production

Intracellular generation of ROS was quantified using 5-(and 6)-carboxyl-2′,7′-dichlorodihydro fluorescein diacetate (DCFDA; Molecular Probes, Eugene, OR, USA). For the assay, the HaCaT cells were cultured overnight in 6-well plates and then treated with 20 Gy of radiation in the presence or absence of EC (100 µM) for 24 h. The cells were incubated in the dark with 10 µM DCFDA in serum-free medium for 10 min at 33°C. An oxidative burst (hydrogen peroxide, H_2_O_2_) was detected using a FACScan flow cytometer (BD Biosciences) with excitation and emission settings at 488 and 530 nm, respectively.

### Western Blot Assay

The HaCaT cells were pre-treated with EC (100 µM) for 1 h, and then the cells were washed twice with cold PBS. Subsequently, the cells were irradiated with 20 Gy or not.Total proteins were extracted using the ProteoExtract® Subcellular Proteome Extraction Kit (Calbiochem, La Jolla, CA, USA) following the manufacturer’s instructions. Proteins were processed as described previously [22]. All primary antibodies were purchased from Cell Signaling Technology (Danvers, MA, USA) and antibody dilution concentrations were 1: 1000.

### Animal Study

Thirty two female, 6-week-old, 145–160 g (mean 152±3.5 g) Sprague-Dawley rats (Samtaco, Osan, Korea) were maintained in the central animal laboratory for at least 1 week. The animals were housed in small groups of three animals per cage and allowed free access to water and food. The temperature was maintained at 21±1°C and lights were turned on from 8∶00 AM to 8∶00 PM. This study was approved by the Committee for Ethics in Animal Experiments, Ajou University School of Medicine (IACUC number: AMC115). The rats were randomly divided into four groups (n = 8 per group). The control group did not receive any radiation or EC. The EC alone group (EC group) was treated with EC (Sigma-Aldrich). The radiation+epicatechin group (RT+EC group) was treated with EC after irradiation. The radiation alone group (RT group) received radiation and no further treatment. The rats in the RT+EC and RT groups were sedated using an intraperitoneal (IP) injection of 3.125 mg/kg tiletamine, 3.125 mg/kg zolazepam, and 11.5 mg/kg xylazine hydrochloride. The rats were placed and restrained in the prone position on an acryl plate. Radiation was restricted to the oral cavity to spare the rest of the body (superior border of radiation: transverse line between lower eyelid and lower margin earlobe; posterior border of radiation: imaginary vertical line of earlobe). A single 30 Gy dose was delivered by opposed photon beams at a rate of 2 Gy/min bilaterally at a distance of 100 cm from the source to the axis using the LINAC, 6MV (21EX; Varian). Tissue-equivalent material was placed on the irradiated regions to ensure a uniform distribution of the prescribed dose. Rats in the RT+EC group received oral treatment with EC using a feeding device each day at 9∶00 AM, 1∶00 PM, and 6∶00 PM (2 mM, 100 µL/dose, total 300 µL/day) for 23 days after irradiation.

Euthanasia of rats was expected if rats demonstrated the conditions listed below, whether the animal had been manipulated or not. (1) Weight loss: loss of 20–25%. (2) Inappetance: complete anorexia for 24 hours and partial anorexia (less than 50% of caloric requirement) for 3 days. (3) Weakness/inability to obtain feed or water: Inability or extreme reluctance to stand which persists for 24 hours, assuming that the rat has recovered from anesthesia. (4) Moribund state: measured by a lack of sustained purposeful response to gentle stimuli (example of purposeful response- weak attempt to get up; if animal is on its side, attempts should be asymmetrical in nature).

### Assessment of Radiation Damage in Rats

All animals were monitored daily to examine status of the oral cavity, amount of oral intake, weight, and survival. To evaluate the histopathologic changes in the tongue and buccal mucosa, one rat in each group was sacrificed on the 4^th^ and 9^th^ day after irradiation, respectively. Mucosa of the tongue and buccal mucosa was harvested from rats that died during the experiment and sacrificed at end of the experiment. The tongue and both buccal mucosa were exposed and photographed. Tissue samples of tongue and buccal mucosa were fixed in 10% buffered formalin and embedded in paraffin blocks. Each paraffin-embedded specimen was sectioned at a thickness of 5 µm. The paraffin was removed by immersion in graded series of ethanol (from 100 to 70%), and each section was then stained with a hematoxylin-eosin solution and observed with an Olympus BX-51 microscope (Olympus, Tokyo, Japan). We also analyzed TUNEL staining as described below.

### TUNEL Staining in Rats

Apoptosis of oral mucosa was determined by the TUNEL method using an *in situ* detection kit (TACS 2 TdT DAB kit; Trevigen, Gaithersburg, MD, USA) according to the manufacturer’s instructions. The tissue slides were then immersed in quenching solution and 1× terminal deoxynucleotidyl transferase (TdT) labeling buffer and incubated in brominated nucleotide (BrdU)-labeling reaction mix. Visualization of apoptotic cells was achieved by incubating the sections in diaminobenzidine/hydrogen peroxide solution. Finally, the slides were counterstained with methyl blue solution, and the stained slides were observed and photographed using light microscopy.

### Immunohistochemical Analysis in Rats

For immunohistochemistry study, DAKO immunohistochemistry kit (DAKO LSAB Universal K680, Carpinteria, CA, USA) was used according to the manufacturer’s instructions. Specimen sections of 5 µm were deparaffinized in xylene and rehydrated through a gradual concentrations of ethanol. The endogenous peroxidase was blocked with 3% hydrogen peroxide for 5 min at room temperature following PBS washes. Nonspecific binding was blocked with 1% bovine serum albumin for 1 h. Anti-NOX-3 antibody (Sigma Aldrich) was added to the slides, and incubation proceeded for 1 h. After repeated washes with PBS, the sections were incubated with biotinylated secondary rabbit antibody for 1 h containing horseradish peroxidase. Finally, the sections were stained in a freshly prepared substrate solution (3 mg of 3-amino-9-ethylcarbazole in 10 ml of sodium acetate buffer pH 4.9, 500 µL of dimethylformamide, 0.03% hydrogen peroxide) for 10 min.

### Statistical Analyses

The Student’s t test and one-way ANOVA were used for the statistical analyses of the *in vitro* data. *In vivo* study, all values were expressed as mean ± standard deviation and statistical analysis was performed by the Kruskall-Wallis Test and the Mann-Whitney U Test. The survival rates were evaluated using the Kaplan Meier method (SPSS, version 17, Chicago, IL, USA). A *P*-value <0.05 was regarded as statistically significant.

## Results

### Pretreatment with EC Increased Viability of Irradiated HaCaT Cells

**Figure 1:**
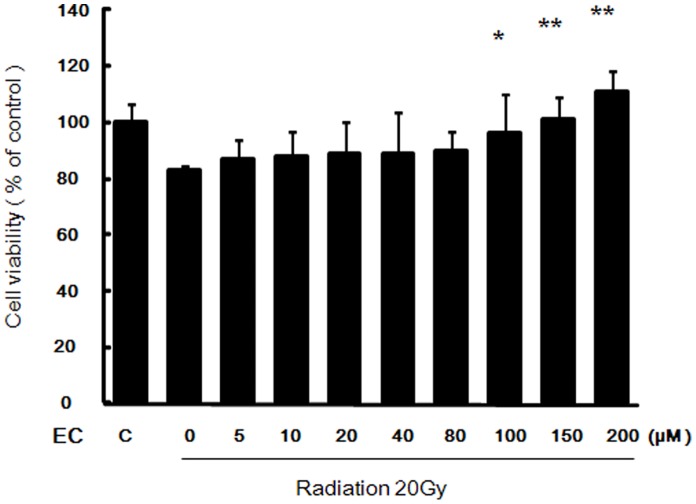
Effect of epicatechin on viability of the HaCaT cells after treatment with radiation. The HaCaT cells were exposed to a single dose of radiation (20 Gy), various concentrations of EC (0–200 µM) or radiation plus EC. At 5 days interval after irradiation, cell viability was measured by MTT assay. The data represent the mean ± SD of three independent experiments. * *p*<0.05, ** *p*<0.01, *** *p*<0.001, compared to radiation alone.

As shown in Figure 1, radiation decreased the viability of the HaCaT cells, but EC significantly protected the HaCaT cells from radiation induced cytotoxicity in a dose-dependent manner.

### EC Protected HaCaT Cells Against Radiation-induced Apoptosis

**Figure 2:**
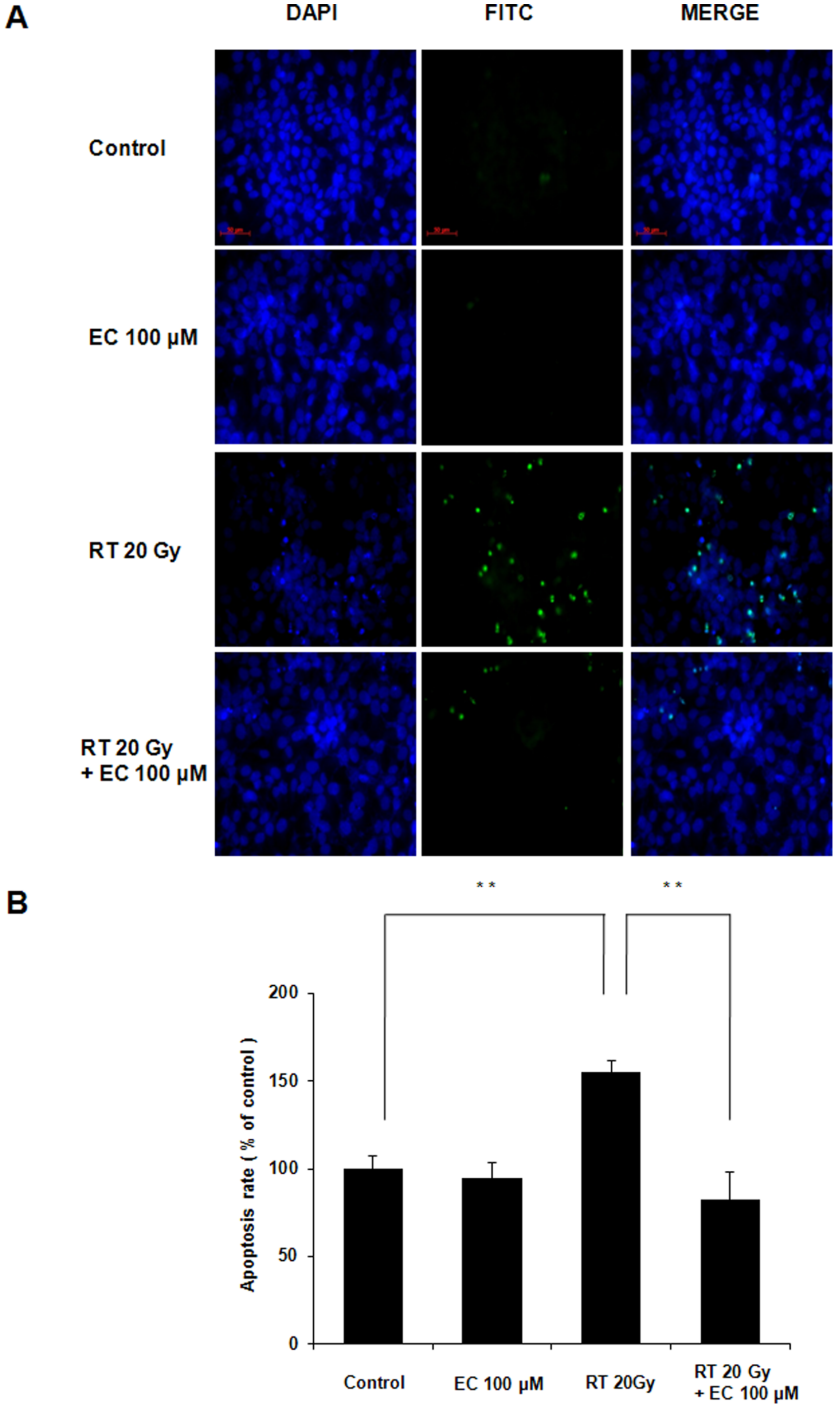
Protective effect of epicatechin against radiation toxicity in HaCaT cells. (A) TUNEL assay of treated HaCaT cells. EC reduced radiation-induced apoptosis of HaCaT cells. Scale bar = 50 µm. (B) Quantification of percentage of apoptosis seen in flow cytometry of Annexin V-FITC and PI double stained HaCaT cells. The data represent the mean ± SD of three independent experiments. ** *p*<0.01, by one-way ANOVA.

Radiation induces HaCaT cell death by apoptosis, so the TUNEL assay and Hoechst 33258 staining were performed to determine if that could be prevented by treatment with 100 µM EC. As shown in Figure 2, EC treatment decreased TUNEL-positive cells. Annexin V-FITC and PI double staining was used to analyze the percentage of apoptotic cells irradiated in the absence or presence of EC (Figs. 2B). Radiation plus EC (100 µM) significantly decreased the number of total apoptotic cells compared to radiation (20 Gy) only.

### EC Inhibited Radiation-induced Changes in the Mitochondrial Membrane Potential

**Figure 3:**
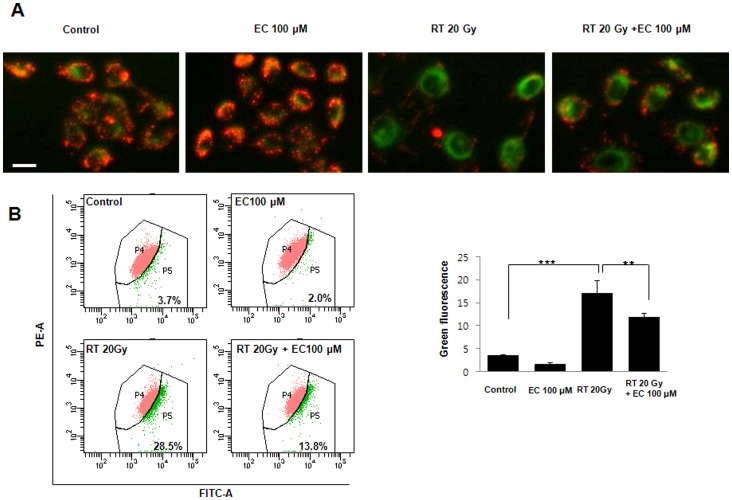
Effect of epicatechin on radiation-induced mitochondrial membrane potential in the HaCaT cells. (A) Cells were irradiated or not (20 Gy) in the absence or presence of EC (100 µM), and stained with JC-1 (red fluorescence). Increase of green fluorescence in irradiated cells suggest loss of MMP. (B) The MMP change was objectively measured using the flow cytometry FACScan. The data represent the mean ± SD of three independent experiments. ** *p*<0.01, *** *p*<0.001. Scale bar = 50 µm.

As shown in Figure 3, in the control cells, a high MMP was maintained, as indicated by the predominantly red fluorescence of the JC-1 dye. However, radiation treatment decreased the green cell fluorescence indicating a loss of MMP and mitochondrial damage. Treatment with EC inhibited radiation-induced changes in the MMP and cell morphology (Fig. 3).

### EC Inhibited Intracellular ROS Generated by Radiation

**Figure 4:**
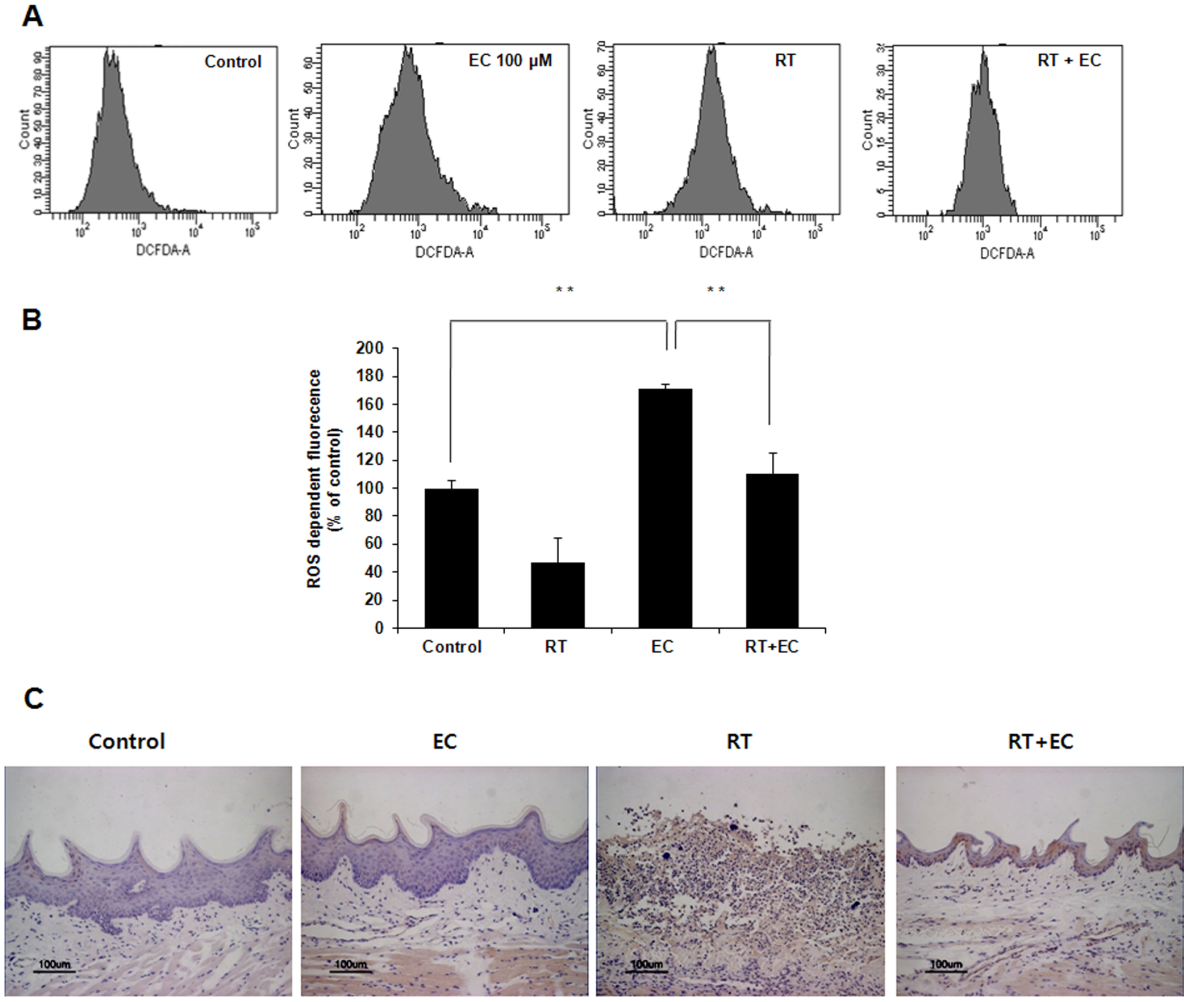
Effect of epicatechin on radiation-induced intracellular ROS generation in HaCaT cells and radiation-induced NOX3 expression in rat oral mucosa. (A) Intracellular ROS generation was measured in HaCaT cells treated with 20 Gy of radiation in the presence or absence of EC (200 µM). The level of intracellular ROS was measured by FACScan flow cytometry using the peroxide sensitive fluorescent probe, DCFDA. (B) The results (mean ± SD) were calculated as a percent of the control group (not exposed to radiation). The data represent the mean ± SD of five independent experiments. ** *p*<0.01. (C) Immunohistochemistry of NOX-3 in control, radiation alone, EC alone and radiation plus EC treated rat oral mucosa sections from each experimental group were stained with anti-NOX-3 antibody. Scale bar: 100 µm.

We examined the effects of EC on radiation-induced ROS generation. Irradiation significantly increased the intracellular ROS generation. EC significantly reduced radiation-induced the generation of intracellular ROS generation (Fig. 4A, B), suggesting that increased ROS generation may be involved in radiation-induced HaCaT cell apoptosis. To demonstrate the effects of EC on radiation-induced ROS generation *in vivo* rat model, we performed an immunohistochemistry study for NOX. In immunohistochemistry study, radiation markedly induced the expression of NOX-3 protein in the oral mucosa of rat. However, NOX-3 protein was significantly decreased in the oral tongue and buccal mucosa in rat treated with both EC plus radiation compared to rat treated with radiation alone. (Fig. 4C).

### Inhibition of MAPK Activity by Epicatechin Rescued the HaCaT Cells from Radiation Induced Cytotoxicity

**Figure 5:**
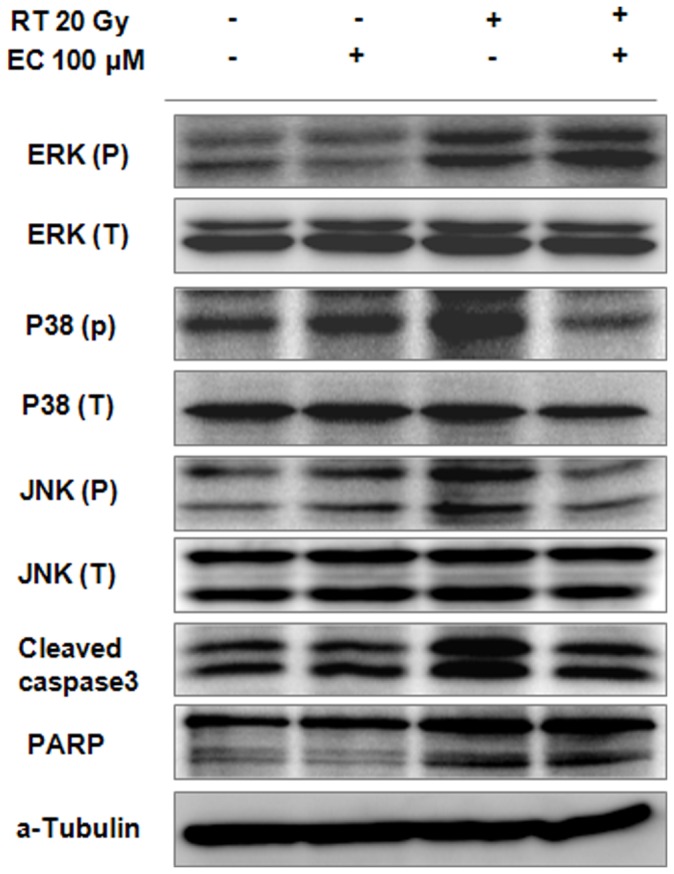
Inhibition of radiation-induced phosphorylation of MAPKs and caspase-3 dependent pathway by epicatechin treatment in HaCaT cells. The cells were pretreated with 200 µM of EC followed by the addition of 20 Gy radiation. Immunoblot of treated HEI-OC1 cells stained with antibodies against p38, JNK, ERK, and cleaved caspase-3.

To clarify the associated mechanism regarding the activity of radiation and EC, we evaluated the radiation and EC-induced changes on protein expression of the MAPK pathway and the apoptotic signal pathway. The effects of EC treatment on the expression of p38, JNK, and cleaved caspase-3 were investigated by Western blot analysis. A representative Western blot is shown in Figure 5. The results obtained confirmed increased expression of p-JNK, p38, and cleaved caspase-3 after radiation treatment. These results suggest that EC blocked radiation-induced apoptosis via down-regulation of JNK and p-38 in the HaCaT cells.

### EC Restored the Oral Intake, Weight Loss, and Survival Rate of Irradiated Rats

**Figure 6:**
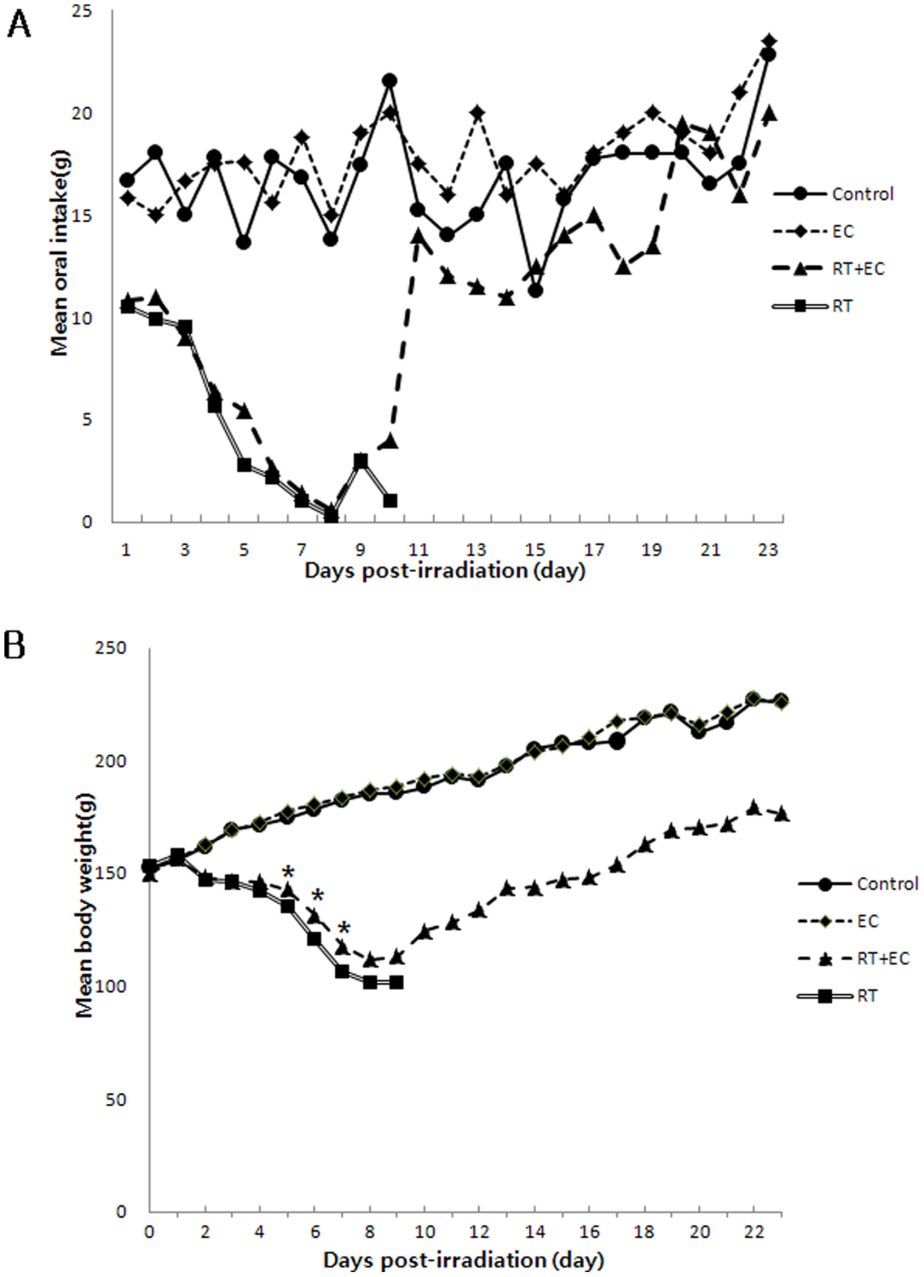
Effect of epicatechin on oral intake and body weight of irradiated rats. (A) Effect of EC on rat oral intake. On the 1^st^ day after irradiation, the oral intake of irradiated rats began to decrease. The oral intake in the rats in the RT+EC group started to increase on the 9^th^ day after irradiation and was markedly increased on the 11^th^ day. The mean oral intake of the RT+EC group was similar to that of the control group between the 11^th^ and 23^rd^ day (*P = *0.09, Mann-Whitney U test). (B) Effect of EC on rat body weight after irradiation. On 2^nd^ day after irradiation, the body weight of irradiated rats began to decrease. The RT+EC group displayed a statistically significant lower weight loss than the RT group on the 5^th^, 6^th^ and 7^th^ day (*p<0.05). The rats in the RT+EC group started to gain weight on the 10^th^ day and reached their original weight on the 17^th^ day. Weight gain velocity was not significantly different as compared with the control group (*p = *0.75).

On the 1^st^ day after irradiation, the oral intake of the irradiated rats began to decrease and declined until day 8. The mean oral intake between irradiated groups was not significantly different until day 10. The oral intake of the rats in the RT+EC group started to increase on day 9 and was markedly increased on day 11. The mean oral intake of the RT+EC group was not statistically significantly different to that of the control group between 11^th^ and 23^rd^ day. The mean oral intake of the control group was similar to that of the EC group during the experimental period (Fig. 6A).

The body weight of irradiated rats started to decrease on post-irradiation 2^nd^ day and significantly decreased between 2^nd^ and 9^th^ day as compared with the control and EC groups. The rats in the RT group did not gain weight before death. The RT+EC group showed a statistically significant less weight loss than the RT group on 5^th^, 6^th^ and 7^th^ days (* *p*<0.05). The rats in the RT+EC group began to gain weight on 10^th^ day, reached their original weight on 17^th^ day, and continuously gained thereafter. The weight gain velocity of the RT+EC group was not significantly different as compared with the control and EC groups (Fig. 6B).

In the RT group, two rats died on 8^th^ day, two rats on 9^th^ day, and the other on 10^th^ day. In the RT+EC group, one rat died on 9^th^ day, and two rats died on 10^th^ day. The survival rates of the control group, EC group, RT+EC group, and RT group on day 23 were 100% (6/6), 100% (6/6), 50% (3/6), and 0% (0/6), respectively (Table 1).

**Table 1:** Survival rate and time of death of each group of rats.

Groups	No. of surviving rats/(No. of sacrificed rats)/No. of total rats	Number of deaths						
		Days post-irradiation						
		1∼3	4	5∼7	8	9	10	11∼23
Control	6/(2)/8	0	0 (1)	0	0	0 (1)	0	0
EC	6/(2)/8	0	0 (1)	0	0	0 (1)	0	0
RT+EC	3/(2)/8	0	0 (1)	0	0	1 (1)	2	0
RT	0/(2)/8	0	0 (1)	0	2	2 (1)	2	

Abbreviations: EC group = epicatechin alone group, RT+EC group = radiation+epicatechin group, RT group = radiation alone group, (): One rat each in all groups was sacrificed for histopathologic evaluation on day 4 and 9, respectively.

### EC Inhibited Histopathologic Changes of Oral Mucosa by Irradiation in Rats

**Figure 7:**
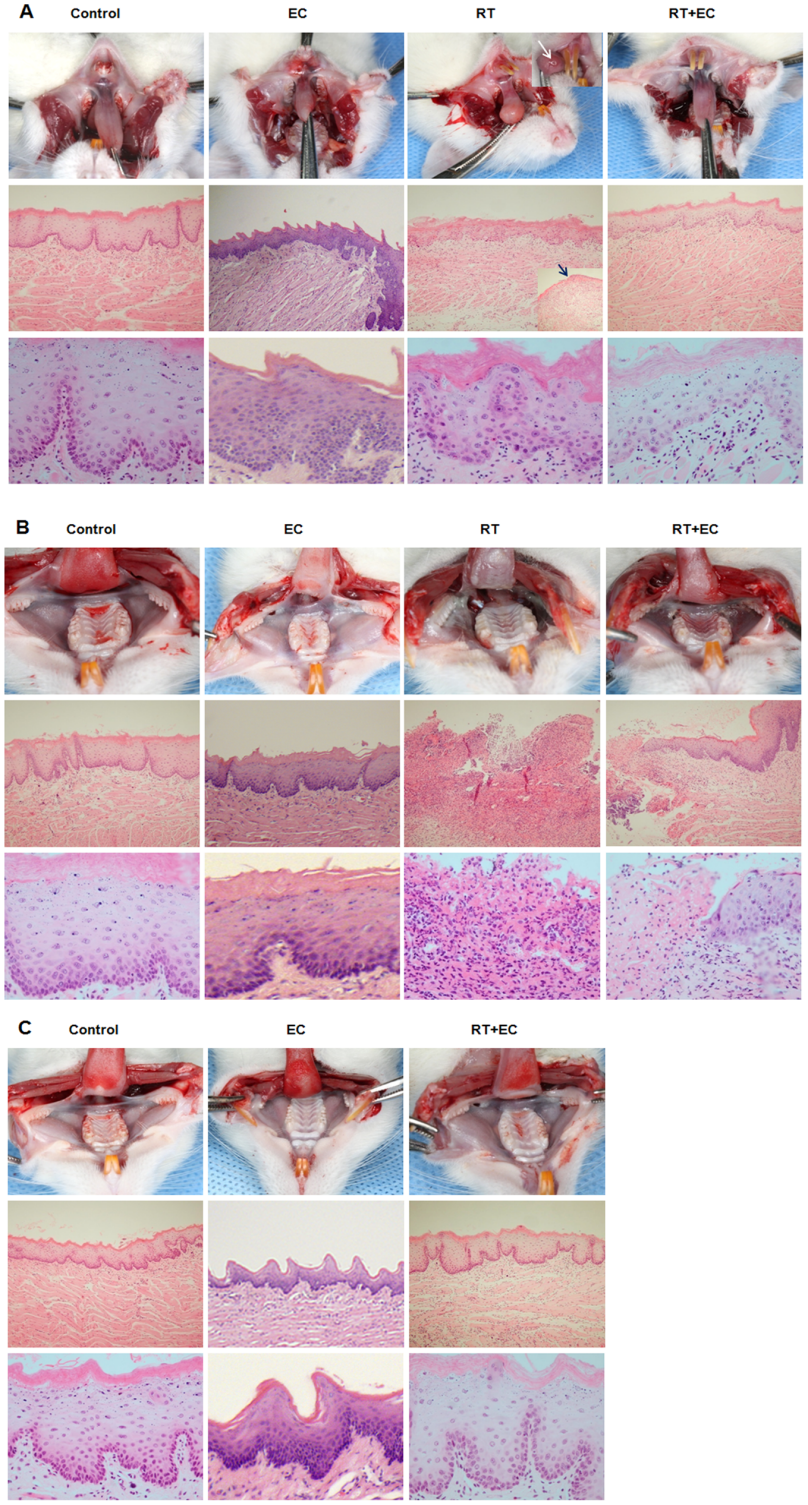
Effect of epicatechin on histopathologic findings of irradiated rats. (A) Gross photographic images and histopathologic images of oral mucosa on the 4^th^ day after irradiation. Each paraffin-embedded specimen was sectioned at a thickness of 5 µm. (H&E staining: x100 and x400). Tongue tip ulceration (arrow). (B) The gross photographic images and histopathologic images (H&E staining: x100 and x400) of oral mucosa on the 9^th^ day after irradiation. (C) The gross photographic images and histopathologic images (H&E staining: x100 and x400) of oral mucosa on the 23^rd^ day after irradiation.

One rat in each group was sacrificed for histopathologic evaluation of the oral mucosa on post-irradiation 4^th^ and 9^th^ day. All remaining rats were examined after death during the experiment or, if still alive on 23^rd^ day, were sacrificed and analyzed. In the control and EC groups, histopathologic findings of oral mucosa on 4^th^, 9^th^, and 23^rd^ days were similar. On 4^th^ day after irradiation, oral mucositis was not grossly evident, except for ulceration of the tongue tip in the RT group. However, histopathologically, the buccal mucosa of the RT group showed mild submucosal edema with inflammatory cell infiltration and tongue tip ulceration. High magnification views showed partial nucleopleomorphism and extracellular edema in the buccal mucosa of the RT group (Fig. 7A). However, in other groups, we could not find typical abnormal findings. On post-irradiation 9^th^ day, grossly intact anterior buccal mucosa and mild ulceration in the posterior buccal mucosa and tongue were detected in the rats of the RT+EC group as compared to the RT group, which showed severe ulceration and necrosis in the whole tongue and oral mucosa. Histopathologically, the buccal mucosa of the RT+EC group showed ulceration with epithelial change in adjacent mucosa, and the buccal mucosa of the RT group was severely ulcerated with necrotic inflammatory exudates (Fig. 7B). On 23^rd^ day after irradiation, the tongue and buccal mucosa of the RT+EC group were similar to those of the control group. Healed mucosa was observed in hematoxylin-eosin stained tissue and little difference was seen between the RT+EC group and the non-irradiated control and EC groups (Fig. 7C).

### EC Reduced Radiation-induced Apoptosis in Rat Oral Mucosa

**Figure 8:**
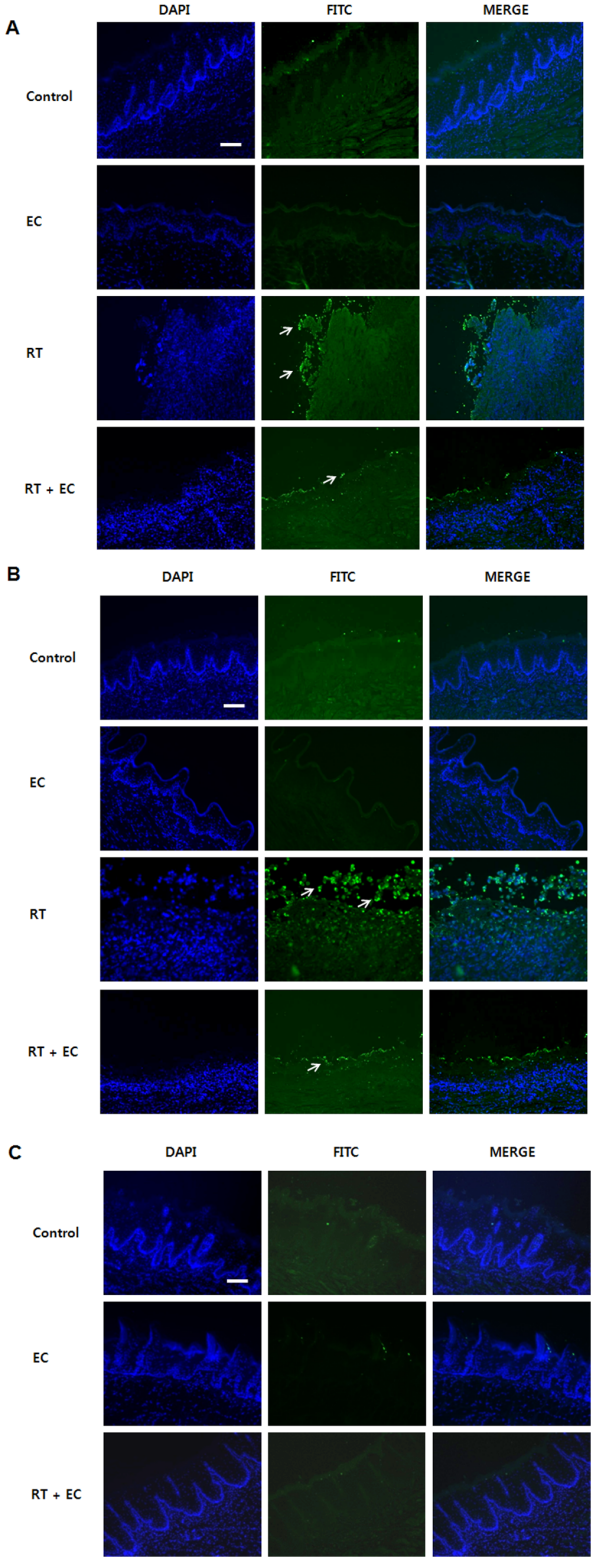
Protective effect of epicatechin against radiation in rat oral mucosa, as indicated by TUNEL staining for apoptosis. (A) The 4^th^ day after irradiation, (B) The 9^th^ day after irradiation, (C) The 23^rd^ day after irradiation. TUNEL staining of rat mucosa revealed EC treatment significantly decreased radiation-induced TUNEL-positive cells (arrow). Scale bar = 50 µm.

To determine the nature of cell death, TUNEL staining was carried out on all four groups. TUNEL-positive cells were not observed in any of the oral mucosa from rats in the non-irradiated control and EC groups. On post-irradiation 4^th^ day in the RT group, weak TUNEL-positive cells were evident around the ulcerated region of the tongue tip (Fig. 8A). In contrast, the oral mucosa from radiation-treated rats showed marked TUNEL-positive labeling of a variety of cell types within the irradiated mucosa on post-irradiation 9^th^ day (Fig. 8B). However, TUNEL analysis revealed the reduction of TUNEL-positive cells by EC treatment. On post-irradiation 23^rd^ day, TUNEL findings of the RT+EC group were similar to those of the control and EC groups (Fig. 8C).

## Discussion

The exact pathophysiology of radiation-induced oral mucositis is not been fully known. The onset of oral mucositis is a biologically complex process that has only been partially elucidated at the molecular level [23]. Radiotherapy and chemotherapy are known to lead to the generation of ROS that activate several signaling pathways in the submucosa and epithelium [23], [24]. These activated pathways lead, in turn, to abrogate epithelial cell renewal, spur apoptosis, increase atrophy, and trigger ulcer formation. Based on this ROS-dependent mechanism of radiation-induced oral mucositis, various antioxidant strategies can be considered. [25] We speculated that EC, which is well-known anti-oxidative phytochemical, could be a possible solution for ameliorating radiation induced oral mucositis. We previously reported about the otoprotective effect of EC against radiation and cisplatin through the reduction of ROS generation [26]–[28]. In the present study, we intended to investigate the protective effect of EC against radiation-induced cellular damage *in vitro* in the HaCaT cells and *in vivo* in a rat model.

In this investigation, we revealed that radiation-induced apoptosis of the HaCaT cells was dose-dependently prevented by EC treatment. EC reduced the intracellular ROS generation which induced by irradiation. These results indicate that radiation-induced cellular toxicity is closely connected with intracellular ROS generation and EC may ameliorate this toxicity by diminishing oxidative cellular damage. Regarding the associated mechanism, JNK and p38 in MAPKs were activated in irradiated cells. In addition, we demonstrated the possibility of EC to inhibit radiation-induced oral mucositis in a rat model. Radiation-induced oral mucositis decreased oral intake, weight, and survival rate, but these were significantly restored by oral administration of EC. Histopathologic changes and TUNEL positive cells induced by irradiation were significantly decreased in the oral mucosa of the EC-treated rats.

Green tea is a worldwide popular beverage and its main ingredients, green tea polyphenols have been reported to exert anticancer, antiinflammatory, antibacterial and antioxidative properties [29], [30]. Tea polyphenols may exhibit antioxidant effects through direct radical scavenging, down regulation of radical production, elimination of radical precursors, metal chelation, or elevation of endogenous antioxidants [31]. Furthermore, a growing body of evidence suggests that polyphenols regulate cell signaling and affect disease progress [31]. One of the important molecular mechanisms associated with the cellular protective activities of polyphenols are MAPK kinases. Epigallocatechin gallate and EC were reported to reduce the phosphorylation of MEK1/2, ERK1/2, and c-Jun [32], [33]. Schroeter *et al.* reported that EC and its metabolite 3′-O-methyl-(−)-EC activated ERK in neuronal cells, as well as the ERK downstream target CREB [34]. In our previous investigation, EC significantly reduced the radiation-induced expression of p-JNK, p-ERK, in cochlear organs of Corti-derived cell lines, HEI-OC1 [28]. EC also protected the auditory organ by attenuating cisplatin-induced ototoxicity through inhibition of ERK [27]. In this investigation, EC treatment significantly reduced the ROS generation and expression of p-JNK and p38 increased by irradiation. The protective effect of EC on radiation-induced cytotoxicity might be related to the inhibition of MAPK expression.

In the present study, staining with the fluorescence dye JC-1 revealed that radiation induced the loss of MMP, which is a marker of mitochondrial membrane integrity. Mitochondria are known to be a producer of ROS as well as the primary target of ROS induced cytotoxicity. Therefore, there is an inextricable connection between mitochondrial damage and intracellular ROS generation, and consequent oxidative stress [35]. Regarding the mitochondrial protection efficacy of EC, Prince *et al* reported that pretreatment with EC could prevent cardiac mitochondrial damage in myocardial infarction [36]. Our results suggested the possible connection between the radio-protective effect of EC and mitochondrial protection.

In the present *in vivo* study using a rat model, EC treatment significantly improved the clinical features and histologic findings caused by irradiation. TUNEL staining of rat oral mucosa revealed that EC treatment significantly decreased radiation-induced TUNEL-positive apoptotic cells. The mean oral intake, body weight, and histopathologic findings of the EC group were similar to that of the control group, and none of the EC group died during the experiment. EC showed a considerable radio-protective effect on oral mucositis. In irradiated non–EC-treated rats on the 9^th^ Day, the epithelial layer was severely disrupted, and thick pseudomembrane was formed with dense inflammatory cell infiltration. EC treatment ameliorated the distortion of the epithelial cell layer and reduced the associated inflammation. These histologic findings correlated with the increase of oral intake and body weight of EC-treated rats started on the 9^th^ day. Improvement of oral mucositis may have contributed to the restoration of the oral intake and body weight, eventually leading to the survival of irradiated rats. All non-EC treated rats were not able to survive over 10 days from irradiation; in contrast, 50% of EC treated group survived through study period.

However, the present *in vivo* study was designed as a preliminary animal experiment for evaluation of EC in radiation-induced oral mucositis and an intermediate step toward large-scale animal studies using multi-fraction schemes of irradiation. Even though this study followed previous studies which used large single radiation dose scheme, [15], [37] the small number of rats and the use of a large single dose could be potential limitations. A large single dose using this study may induce tissue changes qualitatively different from that during fractionated radiotherapy in the clinical setting. Therefore, a well-designed animal study using multi-fraction irradiation animal study is warranted.

The advantage of green tea application as a radiation mitigator for oral mucositis is its chemopreventive properties and its well-proven safety. For clinical application of any agent as a radiation mitigator, absolute certainty about the protection factors for tumor and normal tissues is compulsory to avoid unpredictable disease progression or complications. Because EC is a dietary component of green tea which has been ingested by humans for a long time, the ingredients should be safe and non-toxic. The application of tea, as a cancer chemopreventive agent has been thoroughly investigated in the last two centuries. Not only epidemiological, but also laboratory studies have proven the protective effect of tea consumption in the development of certain cancer types [38]–[40]. The molecular mechanisms related to the chemopreventive properties of green tea component are known to be appreciated to the modulating cell signaling pathways such as NF-kB, or MAPK kinases [31]. By regulating signaling pathways, polyphenols could promote cell death and induce apoptosis in premalignant or malignant cells, consequently leading to the inhibition of cancer development or progression.

In conclusion, we demonstrated that EC protected the HaCaT cells *in vitro* from radiation by inhibiting ROS generation, maintaining the mitochondrial integrity, and blocking the MAPKs activation. Moreover, EC is an effective means of reducing oral mucosal side effects and facilitating wound healing after radiation exposure in the oral cavity of rats. Although larger numbers of animals and clinically relevant fractionation schemes are necessary to confirm the effect of EC, these results suggest the possibility of EC to inhibit radiation-induced oral mucositis, a common complication in radiotherapy of head and neck cancers.
